# Chest-Worn Inertial Sensors: A Survey of Applications and Methods

**DOI:** 10.3390/s21082875

**Published:** 2021-04-19

**Authors:** Mohammad Hasan Rahmani, Rafael Berkvens, Maarten Weyn

**Affiliations:** IDLab-Faculty of Applied Engineering, University of Antwerp-imec, Sint-Pietersvliet 7, 2000 Antwerp, Belgium; mohammad.rahmani@uantwerpen.be (M.H.R.); rafael.berkvens@uantwerpen.be (R.B.)

**Keywords:** accelerometry, seismocardiography, heart rate, respiration rate, activity recognition, posture analysis, pedestrian dead reckoning, voice activity detection, swallow detection, context retrieval

## Abstract

Inertial Measurement Units (IMUs) are frequently implemented in wearable devices. Thanks to advances in signal processing and machine learning, applications of IMUs are not limited to those explicitly addressing body movements such as Activity Recognition (AR). On the other hand, wearing IMUs on the chest offers a few advantages over other body positions. AR and posture analysis, cardiopulmonary parameters estimation, voice and swallowing activity detection and other measurements can be approached through chest-worn inertial sensors. This survey tries to introduce the applications that come with the chest-worn IMUs and summarizes the existing methods, current challenges and future directions associated with them. In this regard, this paper references a total number of 57 relevant studies from the last 10 years and categorizes them into seven application areas. We discuss the inertial sensors used as well as their placement on the body and their associated validation methods based on the application categories. Our investigations show meaningful correlations among the studies within the same application categories. Then, we investigate the data processing architectures of the studies from the hardware point of view, indicating a lack of effort on handling the main processing through on-body units. Finally, we propose combining the discussed applications in a single platform, finding robust ways for artifact cancellation, and planning optimized sensing/processing architectures for them, to be taken more seriously in future research.

## 1. Introduction

The use of wearable sensors has been significantly increased over the past years [1]. Early motivations to produce such wearables are found in medical applications. Screening patients with heart, Parkinson’s and epilepsy diseases from home to enable early detection of cardiac, Parkinsonian and seizure attacks are a few examples of such motivations [2]. Later advances in dimensions, performance, variety and affordability of wearable electronics let these devices address even wider areas of interest. Today, wearable sensors provide solutions to many sectors from healthcare and wellness to entertainment, security and so on. They are available in several wearable forms such as wristbands, chest-straps and armbands with various sensors embedded in them: temperature and humidity sensors, microphones and image sensors, pressure and force sensors, motion and inertial sensors and a wide range of biomedical sensors, to name a few [3].

Inertial Measurement Units (IMUs) are among the most common sensors used in wearable devices. They may consist of accelerometers, gyroscopes and magnetometers to give a comprehensive sensing from the inertial status of the moving object. Studies indicate that accelerometers are the most frequently implemented IMUs in wearable devices as well as the most frequently addressed sensors by mobile apps [4].

IMUs suggest various applications based on their use parameters. From a wearable device perspective, an IMU provides a low-cost, low-power, locally-computed and thus privacy-respecting sensing of movements that can lead to a continuous tracking of speed, position and attitude of the person [5]. Advances in signal processing and Machine Learn- ing (ML) techniques as well as the production of light-weight Micro-electro-mechanical System (MEMS)-based inertial sensors have broadened the application domains of these sensors. These application domains cover a wide range of movement signals from high-speed running to weak heart-induced vibrations [6,7]. These applications are defined for wearable inertial sensors depending on the placement of an IMU on the body and its targeted moving object from which inertial information is sensed.

Wrists may be referred to as the most frequent hosts for smart wearables, and hence, wrist-worn sensors serve as the most popular wearable devices in the market [3]. The availability of a number of informative body signals such as Photoplethysmogram (PPG), Electro-dermal Activity (EDA) and temperature on the wrist as well as the fashionability of the wristbands have helped these wearables gain relatively higher attention from the market. On the other hand, one can hardly name a smart wristband that does not include an IMU. These IMUs enable the smart wristbands to not only track activities but also measure sleep parameters such as sleep time and efficiency [8,9,10]. However, wristbands face several limitations when it comes to measuring specific types of activities or body parameters.

To make a decision for placement of the wearable inertial sensor, one can roughly categorize the body positions based on either of the following criteria:

1. The type, amount and range of the movements that the sensor measures.
2. The availability of the aimed signals at the selected position.

The former approach may be the best to consider for the purpose of applications, where the movement of a body section plays the main role as in Activity Recognition (AR), while the latter is best to inspect when it comes to measuring a biomedical parameter from the body. Choosing the chest as the main focus of this survey should be investigated within these two scopes. The next two paragraphs, discuss the chest based on the two mentioned criteria, respectively.

To analyze details of some activities, such as running or tooth brushing, ankles and wrists seem to be the best choices, respectively. However, chest and waist would serve better in applications where the body’s center of mass is relevant. As a practical example, Altini et al. [11] used five accelerometers worn on different parts of the body and showed that the one on the chest is the most precise for estimation of Energy Expenditure (EE), while the one on the wrist is the worst. Elbasiony and Gomaa [12] reviewed studies on IMU-based AR topics and concluded that while wrist worn sensors can more efficiently classify non-ambulation activities (such as hair combing, eating, etc.), chest- and waist-mounted sensors show better performance for ambulation activities (such as running, jumping etc.). On the other hand, among the advantages that add value to the chest-worn wearables, being in close contact with the heart has always been on top.

Seismocardiography (SCG) is the act of analyzing vibrations of the chest wall induced by the heart activity [13,14]. These vibrations are always modulated by the lungs’ motions, making SCG a rich information source for cardio-respiratory analysis [14]. Although the definition provided for SCG is wide enough to cover both rotational and axial vibrations, some references tend to use a different term for analysis of the rotational components measured by a gyroscope, i.e., Gyrocardiography (GCG) [15,16,17]. Within the scope of such papers, SCG only refers to the axial vibrations usually captured using an accelerometer. This survey, however, uses the term SCG to refer to the use of an IMU aiming to capture and analyze the cardio-respiratory originated vibrations.

**Figure 1:**
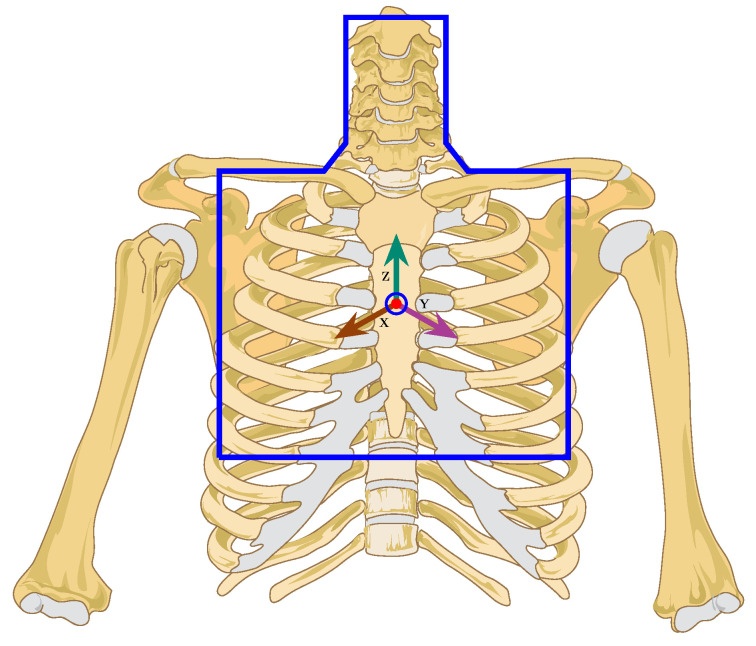
Area of interest of this survey.

Despite all the efforts done using the chest-worn IMUs, we noticed the lack of a comprehensive survey on the applications and methods associated with these sensors. This could attract more attention to the current capabilities of such wearables, highlight the existing challenges to be solved and address the undiscovered potential in this area. This survey paper aims to identify and introduce a wide range of application domains that can be addressed through a chest-worn IMU. It is very interesting to see how much we can perceive from a single simple sensor once we find the correct body area to put it on. The chest is the key area as it is an intersection of a bunch of body signals, i.e., heart, respiration, voice, swallowing and of course the movements of the whole body. Figure 1 shows the area of interest of our paper.

Table 1 lists the most recent published reviews relevant to the current work to highlight the difference. Cosoli et al. [18] systematically reviewed the latest wrist-worn and chest-strap wearable devices to analyze their accuracy and metrological characteristics. Their focus was mainly on finding validation standards for their analysis. They also limited their scope to the devices for activity monitoring. Taebi et al. [7] reviewed the recent advances in SCG up to 2018. They analyzed the measurement sensors and their placement as well as the methods for different stages of the signal processing. Since SCG is a part of the current survey, we have mainly put our focus on the SCG papers not included in their work, i.e., published after 2018. However, to include enough references for both heart and lung parameters analysis, there have been a few SCG studies referenced from before 2018. Kröger et al. [4] reviewed the privacy implications of the accelerometer data. This is a short review with a focus on listing all possible applications of the accelerometer data that can interfere with the privacy of the user.

This survey reviews a total of 57 research articles making use of the chest-worn IMUs and published since 2011 to the end of 2020. These referenced studies are listed in Table A1 in the Appendix A. All the statistics presented in the rest of this paper are based on these referenced studies. The remainder of this survey includes five sections: Section 2 lists existing approaches on possible information to be gathered through chest-worn inertial sensors; Section 3 provides an overview on available methods to measure inertial information from the chest and discusses their validation methods; the existing literature is then further analyzed from the data processing architecture point of view in Section 4; processing stages and the hardware units performing those stages are investigated in this Section; the opportunities to use chest-worn inertial sensors in multi-purpose applications as well as the research challenges and the future directions are discussed in Section 5; and finally, the conclusions are drawn in Section 6.

## 2. Applications

Measurement of movements is the basis of what IMUs provide; however, they suggest much more than AR, thanks to the machine learning techniques supporting them. Kröger et al. [4] provide references on more than twenty different user information categories that can be inferred from accelerometer data. Interestingly, about half of these categories represent behavioral information such as moods and emotions, driving behavior, smoking behavior, etc.

To focus more on chest-worn IMUs, we have categorized the referenced studies into seven distinct areas as listed in Table 2. The current section provides information on these categories.

### 2.1. Seismocardiography

SCG is the measurement and analysis of vibrations on the chest wall induced by the cardio-respiratory activity. Theoretically, there have been several methods to capture these vibrations: IMUs, Laser Doppler vibrometers, Microwave Doppler radars and ultrasound-based methods to name a few [7]; however, the use of IMUs on chest has been the most frequent capture medium in SCG.

New advances in production of low-noise IMUs have improved the quality of SCG recordings. Availability, low power consumption, small dimensions, light weight and low cost of the MEMS IMUs have made them ideal choices for the extension of the SCG applications from clinical diagnostics to real-life monitoring.

For heart monitoring, chest-worn IMUs have shown promising results in detection of the heartbeats [19]. As a next step, analysis of Heart Rate (HR) [20] and Heart Rate Variability (HRV) [16] is reported in the literature. Estimation of Inter-beat Interval (IBI) [21], Aortic valve Opening (AO)-peaks [22], Pre-Ejection Period (PEP) [23] and Left Ventricular Ejection Time (LVET) [24] ands identification of some heart anomalies [25,26] are also conducted via chest-worn IMUs.

Respiratory parameters inferred through chest-worn IMUs, on the other hand, include (but are not limited to) respiration rate [27,28], respiration volume [29], lung capacity [30] and respiration phase [31].

### 2.2. Activity Analysis

Thanks to the abundance of the activity trackers, activity analysis may be the most famous application of the inertial sensors among the others. Automatic recognition and measurement of daily activities, exercises and routines are increasingly getting popular around the world, as suggested by market trends [1].

Since the body’s center of mass is close to the chest, it is an ideal position for hosting AR systems that aim to classify activities such as walking, running, cycling, jumping and pushing up. AR [32,33], EE estimation [34], fall detection [35] and motion tracking [36] are among the applications addressed using chest-worn IMUs.

### 2.3. Posture Analysis

Muting the phone by flipping it faced down or having its screen turned on by just picking it up from a table are familiar applications of IMUs to many people. Posture detection and gesture recognition are traditional use cases of the IMUs frequently experienced while interacting with the smartphones.

IMUs have perfectly added posture analysis capabilities to wearable devices as well. Posture detection, as an important part of sleep analysis, has been implemented via chest-worn IMUs [10,37]. Moreover, postural control for both healthy adults [38] and patients with Multiple Sclerosis (MS) decease [39] has been reported in the literature. As another use case, adding a first step of posture detection has improved AR performance [35].

### 2.4. Localization

Localization is best known with the Global Navigation Satellite System (GNSS); however, satellite-based localization comes with limitations that reduce its effectiveness for indoor positioning and also applications for which power consumption is a critical factor [40]. These applications would be better investigated using methods other than GNSS such as Received Signal Strength Indicator (RSSI) and Pedestrian Dead Reckoning (PDR).

Within PDR, current position is calculated based on measured changes to a previously estimated position. This is best done using an IMU, usually in combination with map matching algorithms. In such cases, knowing the initial condition on the map is a key point for the PDR algorithm to work.

### 2.5. Voice Analysis

Microphones are the main sensors for voice recording; however, there have always been serious privacy and power concerns with them. Consequently, there has always been a tendency to substitute microphones with less privacy-invasive and more low-power sensors or to limit the scope of their usage based on the application. On the other hand, performance of applications such as an Automatic Speech Recognition (ASR) system (which rely on microphones) becomes gradually degraded by environmental noise, which is a challenge to be tackled.

Voice Activity Detection (VAD) is the process of distinguishing between speech and non-speech moments. It improves performance and accuracy of ASR while reducing its power consumption. In noisy environments as well as in multi-speaker setups, VAD leads to a more focused analysis of voice for the ASR. Limiting the scope of microphone usage is another consequence of VAD which is an advantage from both privacy and power points of view.

Use of an accelerometer, typically near the larynx, is a common approach for VAD [41,42]; however, chest-worn accelerometers have also been investigated for this purpose, leading to comparable performance [43,44]. Neck-surface accelerometers have also been used for a diagnostics approach. Mehta et al. [45,46] used a neck-surface accelerometer for measurement of a few vocal functions, namely: time-domain perturbation, spectral harmonicity and cepstral periodicity.

### 2.6. Swallow Analysis

Swallow analysis opens a window to another aspect of physical health, and swallow detection can play an important role as a reflection of healthy behavior. An automated swallow analysis can facilitate measurement of food intake to help monitor diet or treat obesity. On the other hand, screening patients with dysphagia—swallowing difficulties with certain foods or liquids—significantly adds to the importance of swallow analysis.

Swallowing accelerometry is a potential non-invasive method in this field. Use of neck-worn accelerometers alongside PPG is reported for swallow detection [47], and the relation of swallowing vibrations to hyoid bone movement has been investigated in patients with dysphagia [48].

### 2.7. Context Retrieval

Several attempts have been made to retrieve context information through IMUs in general [44,49,50]. However, most of them can also apply to the chest-worn IMUs; we found applications that explicitly retrieved their inertial data from the chest, two of which rely on gait analysis as their first step.

Hashmi et al. [51] retrieved inertial data from a chest-worn smartphone for Emotion Recognition (ER). They used the primary emotions model [52] and reported classification of the six basic emotions—namely: *happiness*, *sadness*, *anger*, *disgust*, *fear* and *surprise*—with an accuracy of 86%.

Riaz et al. [53] conducted an age estimation task based on analysis of normal walk through 6-Degree of Freedom (DoF) IMUs. As the best reported performance, a Root Mean Square Error (RMSE) of 2.94 years was achieved for their estimator under 10-fold Cross Validation (CV) using smartphone’s IMU. Their investigations also pointed out the fact that aging meaningfully affects gait.

**Table 2:** Applications of the chest-worn inertial sensors categorized according to the referenced studies.

Application	Reference
**Seismocardiography**	
Analysis of cardiac parameters	[16,19,20,21,22,23,24,30,36,54,55,56,57,58]
Analysis of respiratory parameters	[28,29,30,36,57]
Mapping SCG to BCG	[59]
Identification of patients with CAD	[25]
Relating SCG to ultrasound images	[60]
Identification of heart failure states	[26]
**Activity Analysis**	
AR	[32,33,35,61,62,63,64,65,66]
EE estimation	[11,34]
Fall detection	[35]
Body motion tracking	[36]
Evaluation of transfer skills of wheelchair users	[67]
**Posture Analysis**	
Postural control for medical approach	[38,39]
Posture detection for sleep analysis	[10,37]
**Localization**	
Indoor positioning with PDR	[68,69,70]
**Voice Analysis**	
Measurement of vocal functions	[45,46]
VAD	[41,43,44]
Voice onset detection	[42]
**Swallow Analysis**	
Swallow detection	[47]
Swallow analysis for dysphagia investigation	[48,71,72,73,74,75]
**Context Retrieval**	
Emotion recognition from gait analysis	[51]
Age estimation from gait analysis	[53]
Age, gender and height estimation from gait analysis	[76]
Detection of mood changes from VAD	[44]
Stress and meditaion detection	[77]
Biometric verification	[78]

## 3. Measurement Methods

In this section, we have focused on the existing methods to measure different data for the application areas provided in Section 2. First, we will look into measurement of the inertial data from the chest. Specifications of the sensors as well as the body points from which the data are measured have been discussed for this purpose. Then, we provide information on the methods applied in combination with the inertial data to validate the research outcomes. The importance of these validation methods can be discussed from two points of view. On the one hand, they suggest existing agreed-upon standards to the researchers who have just started in those areas and on the other hand, their variety show the versatility of the inertial sensors from a new aspect.

### 3.1. Sensor Specifications

All the referenced studies (except one [36]) use commercial off-the-shelf IMUs for measurement of the vibrations and movements. They either have their own electronic Printed Circuit Boards (PCBs) equipped with appropriate inertial sensors or incorporate a research-grade off-the-shelf sensor board package such as Shimmer [79]. Moreover, some studies assessed the use of the smartphone IMUs for their research. As quite available devices, smartphones have become an important part of everyday lives making them interesting choices for the studies. Promising results of such studies not only suggests lower price but also can raise the Technology Readiness Level (TRL).

**Figure 2:**
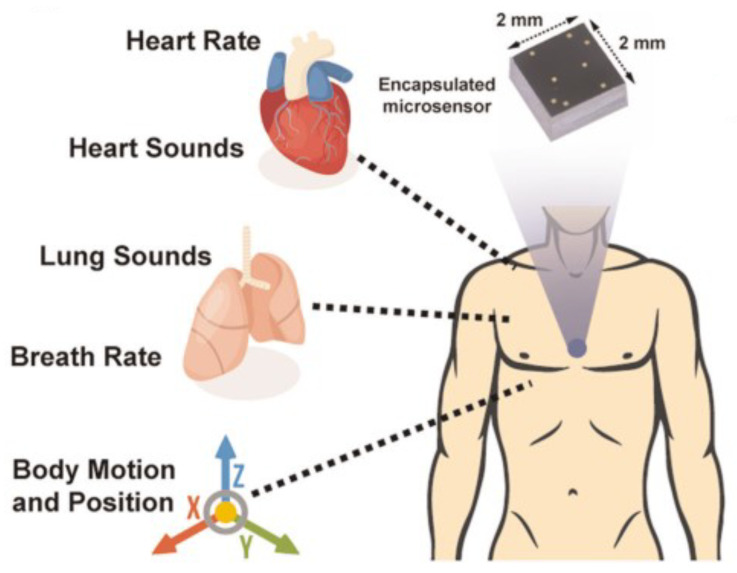
Use of ACM on the sternum to capture cardiopulmonary activity and sounds as well as body motion and position [36].

Gupta et al. [36] aim at encapsulating specifications of an accelerometer and a contact microphone in a single chip to enable simultaneous monitoring of cardiopulmonary vibrations and sounds as well as capturing and analyzing the body motions of the wearer. To address such a wide domain, a carefully designed micro-sensor called an Accelerometer Contact Microphone (ACM) is fabricated to be worn in contact with the sternum (Figure 2). An ACM is claimed to be capable of measuring vibrations from frequencies below 1 Hz (e.g., heart movements) up to 12 kHz (e.g., cardiopulmonary acoustic signals). It is also claims to have a linear response in a wide dynamic range, from 10 μg to 16 g.

The commercial off-the-shelf IMUs used in the referenced studies are listed in Table 3. Looking at the sensitivity of the incorporated sensors reveals that, unsurprisingly, more sensitive sensors are utilized for SCG applications with which smaller vibrations are associated. Despite SCG, applications with higher-range movements such as localization (PDR) and AR do not necessarily utilize very sensitive sensors.

### 3.2. Sensor Placement

The central part of the chest hosts a flat long bone called the sternum or breastbone. The ribs are connected to the sternum, forming the rib cage, which protects the heart and lungs. The sternum is formed of three parts: the *manubrium*, which is the most superior part; the *body*, which is the middle part of the sternum; and the *xiphoid process*, which is the most inferior portion.

**Figure 3:**
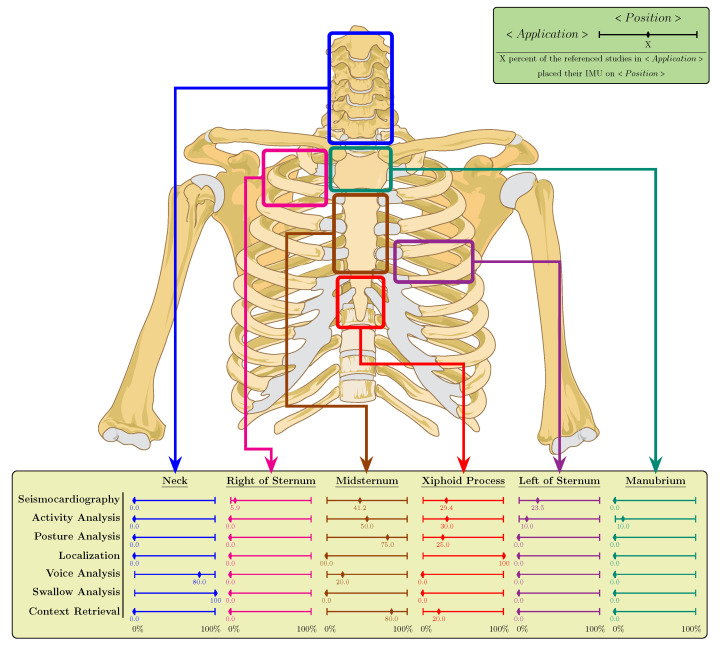
Distribution of the IMUs on chest per application area based on the referenced studies. The percentages are calculated to represent the ratio of the referenced studies in an application area that rely on a specific body site in proportion to the total referenced studies of that application area.

Most of the time, the sternal area is chosen to host the inertial sensors. Figure 3 illustrates the distribution of the IMUs on the body based on the referenced studies. The statistics are given per application area previously were discussed in Section 2. Based on this figure, 100% of the referenced studies in the fields of posture analysis, localization and context retrieval have chosen the two lower parts of the sternum (i.e., the midsternum and the xiphoid process) to place their IMUs on. However, the neck is the preferred position for voice and swallow analysis applications. Moreover, 29.4% of the references in SCG have placed their sensors on the two sides of the sternum. The figure perfectly suggests that if multi-purpose application research was the case, sternum would most likely best serve as an inertial sensor host.

**Figure 4:**
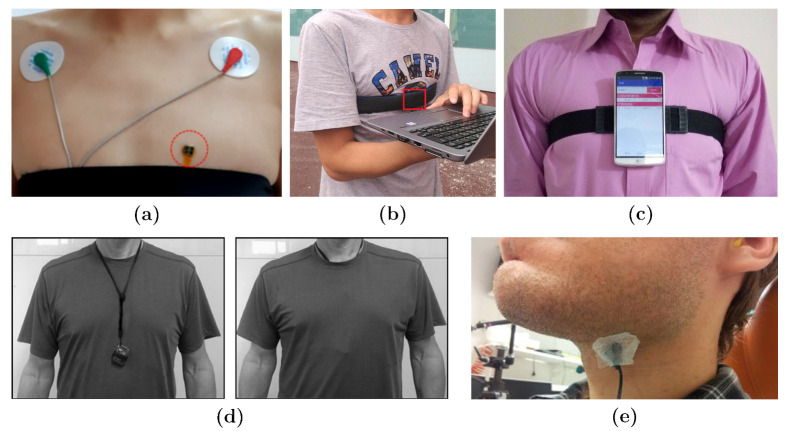
Examples of IMU attachments on the body taken from the referenced studies. (**a**): IMU attached to skin for SCG [54]. (**b**): Use of stretching strap to attach the IMU over clothes for localization [68]. (**c**): Elastic strap used to attach smartphone over clothes for ER [51]. (**d**): Use of a soft nylon necklace over and underneath clothes for EE estimation [34]. (**e**): Attachment of IMU over the skin using adhesive tape for voice analysis [42].

Inertial sensors are usually attached to the chest using elastic straps ([30,54]) or different kinds of adhesives ([29,60]). Applications such as SCG, voice and swallow analysis require the IMU to be in direct contact with the skin; however, wearing the IMUs over the clothes would meet the requirements for other application areas most of the time [10,62,68]. Using a necklace ([34]), fitting the sensor into garments ([33,61]) and holding it in the hand along with the sternum ([39]) are among the other methods used to wear the IMUs in the referenced studies. Figure 4 shows examples of IMU attachments.

### 3.3. Validation Methods

The variety of the applications associated with chest-worn IMUs necessitate various methods to validate the research outcomes. For example, while the decision of an AR classifier may be simply validated by an observer, the HR calculated through SCG would definitely need special measurement devices for validation.

Table 4 lists the validation methods used in the referenced studies versus their application area. It also showcases the usage percentage of uni-/bi-/tri-axial accelerometers, gyroscopes and magnetometers in the studies. This information is given per application area so that a comparison among the use of different IMU types becomes possible. The percentages in the table indicate the ratio of the studies within an application area that utilized a specific type of IMU or validation method.

Based on the table, tri-axial accelerometers are the most frequently used inertial sensors in the referenced studies while magnetometers are the least. Since the term SCG correlates to measuring a wide range of parameters from both the heart and the lungs, there come a lot of validation methods for it (more than any other application area) with Electrocardiography (ECG) as the most frequent one.

## 4. Data Processing

The use of signal processing and machine learning techniques on inertial data has been widely investigated and reviewed in several papers; however, there has been less focus on where in the hardware architecture the data are processed at each stage. Paying too much attention to the processing algorithms has distracted from the fact that the sensing-processing architecture also plays a relatively similar role in determining the usability of the deployed system. This is especially important in the sense that it determines whether or not the hardware design is capable of being implemented out of laboratories or in daily lives.

We covered the measurement methods and hardware in Section 3. In line with the same approach, in this section, we start with investigating different setups used as the sensing-processing architecture in the referenced studies. Next, we present a list of the machine learning approaches used by the referenced studies and provide references for a more comprehensive overview on them. Finally, the publicly available datasets used in the referenced studies are presented.

### 4.1. Sensing-Processing Architecture

Depending on the unit responsible for each processing stage, the following six stages were found to be determinant with respect to the referenced studies: *sensing*, *acquisition*, *transmission*, *storage*, *preprocessing* and *processing*. Moreover, the following distinctive processing units were found to be operational based on the setups: the *on-body* hardware, the *middleware* and the main processing *station*.

The on-body hardware is always responsible for the sensing stage. This originates from the focus of this survey, which is the inertial data taken through chest-worn sensors. The first processing unit that reads the inertial measurement is the acquisition handler. This stage is either done by the same on-body hardware or by a separate middleware, which usually collects data from more than one sensor units. The middleware can either be a Data Acquisition (DAQ) system [59] or a smartphone device [45,46]. The acquired data are then stored by the same data acquirer or transmitted to another unit for storage. The preprocessing and the processing stage is always done by a computer except in two cases. In [65], the processing is handled online using a smartphone as the proccessing station and in [28] part of the preprocessing is done by a smartphone as the middleware. Table 5 shows the setups used in the referenced studies regarding the above-mentioned categorization.

The on-body processing unit is generally one of the following: a sensor platform from those listed in Table 3, a commercial off-the-shelf processor board or a specially designed processing board for the purpose of the study, which consists of a microcontroller. Table 6 lists the on-body processing units on which details were given by the referenced studies. A look over those studies that provided information on the power source shows that, along with battery, USB was used in some studies, which is a reason for stopping the hardware from being used outside laboratory.

The middleware devices used in the referenced studies are listed in Table 7. The first part of the table lists the commercial DAQ systems, of which the related information was given in the referenced studies. In all of these studies, the incorporated inertial sensor has an analog interface, and the signals are read by the Analog to Digital Converter (ADC) channels of the DAQ system. Mehta et al. [45,46] use a smartphone with a principally similar setup of a DAQ system. Their setup uses the handsfree input socket of the smartphone to read the analog vibration signal of a 1-DoF accelerometer. Cesareo et al. [28] used a smartphone to collect the inertial data from their on-body unit through the Bluetooth interface. In their setup, the smartphone preprocesses and stores the data as a middleware.

### 4.2. Machine Learning

As a branch of Artificial Intelligence (AI), ML represents data-driven computer algorithms that improve by learning the patterns found in the data. ML algorithms are categorized into two major classes: supervised learning and unsupervised learning. In supervised algorithms, the machine learns the data by looking at the relationship between the inputs and their resultant outputs; however, in unsupervised algorithms, machine learns the patterns found in the input data to build up its model parameters without having any knowledge about the outputs [81].

Use of signal processing and machine learning techniques on inertial data has been vastly investigated and reviewed in several papers. Table 8 lists the ML methods used by the referenced studies. Based on this table, Regression Models, Support Vector Machine (SVM), k-Nearest Neighbor (k-NN) and Random Forest were among the most frequently used ML algorithms by the studies taking advantage of the chest-worn inertial sensor. For a comprehensive overview of the existing methods of signal processing on inertial data from preprocessing and feature extraction to classification, we would refer the readers to the following papers: [7,81,82,83].

### 4.3. Datasets

Despite the wide range of applications associated with chest-worn inertial sensors, still no relevant benchmark dataset is presented to the researchers. The absolute majority of the referenced studies have collected their own data by recruiting participants of whom the required parameters are measured. However, the five datasets listed in Table 9 were used by a few referenced studies. These datasets are publicly available, and one may access them through the provided references in the bibliography.

## 5. Research Challenges and Future Directions

Several applications are associated with the chest-worn inertial sensors, each of which faces its own challenges: HR detection is highly affected by movement artifacts, fall detection lacks enough real falling data, indoor localization may not solely depend on inertial sensors for a precise reasoning, etc. However, in line with the previous sections, the focus of this section remains on the challenges associated with the chest-worn inertial sensors in general rather than an application-based point of view. Size, power consumption and fashionability of the wearable device would highly affect its capability of daily use. It is of high importance to more strongly highlight these aspects as keys to the user-friendliness of the device for the future work. We will address these issues as well as the challenge of having multiple applications combined in one framework as it would be an interesting research direction with its own limitations and obstacles.

### 5.1. Lack of Well-Acknowledged Benchmark Datasets

Data collection is a critical stage of conducting research. Quality, variety, correctness and amount of data have impacts on the results. Readily available datasets are very important keys not only to facilitate starting a study, but also to prepare benchmark test-beds for various methods to be compared under similar circumstances.

Lack of well-acknowledged benchmark datasets has urged researchers to collect their own data in most of the referenced studies. Therefore, preparation of common comprehensive datasets of chest-worn IMUs for different applications would provide valuable bases for interested researchers.

### 5.2. Robustness and Artifact Cancellation

While several sources affect the quality of the inertial measurements, researchers try to improve the signal quality in different ways. Taking care of the signal quality begins long before the start of the measurements. Use of low-noise electronic elements, robust power and clock design and perfect attachment of the sensor in contact with its target are important keys to improve the quality of the signals for a robust experiment design.

High frequency noise, power line noise, and movement artifacts are the major disruptive factors in quality of the signals. Naturally, there comes high frequency noise associated with the measurements. Such noise is usually defeated by implementing a low-pass filter which is conducted by means of a filter in the preprocessing stage [36,38,56,60]. Band-stop and band-pass filters can address resolving power line noise [56]. More generally different types of band-pass filtering are repeatedly incorporated to limit any out-of-band noise when the frequency band of interest is known [26,37,41,57].

Defeating movement artifact is more critical, especially in applications where the vibration signals of interest are relatively weak which is mainly the case for SCG, voice and swallow analysis. In such cases, a sturdy sensor-skin contact helps reduce the effect of sensor displacement a source of movement artifact. As another workaround, the subjects are often asked to stay motionless during the experiment [23,29,30,59,87]; however, on the one hand, the applicability of this solution in practice is challenging, and on the other hand, it implicitly reduces the extensibility of these studies to real-life implementations. For real-life SCG estimation, a few motionless seconds are said to be enough. Since these motionless seconds take place several times a day, a solution is to use these events to feed SCG system with noiseless inertial measurements from the chest [88]. This solution works for cases such as daily monitoring of the elderly; however, the issue remains challenging for live monitoring of athletes’ cardiopulmonary parameters. Yu and Liu [54] address such challenge by proposing an algorithm for motion artifact removal from SCG signals.

### 5.3. Combined Applications

Few sensors may be found with similar diversity of the application areas as the inertial sensors can bring. This diversity may firstly suggest use of a single chest-worn inertial sensor for simultaneously benefiting from all those applications. However, only few studies used these sensors for multiple simultaneous purposes (only [36] from the referenced studies). The reason may be sought for in the challenges associated with the combined applications.

The application areas investigated in this survey are quite different in the intensity and frequency band of the signals of interest. Applications that deal with weak bio-vibrations require sensors with more sensitivity and less dynamic range, while the applications associated with intense movements need higher dynamic range while being less strict about the sensitivity. However, when using the commercial off-the-shelf IMUs, the dynamic range of the sensor must be set programmatically. The lower the dynamic range set, the higher the sensitivity of the IMU would be. Most of the typical commercial accelerometers suggest the predefined options ±2 g, ±4 g, ±8 g and ±16 g for their dynamic range selection with their highest sensitivity reached when the ±2 g option was selected (e.g., MPU-9250, TDK-InvenSense and LSM6DS3, STMicroelectronics). Similar conditions apply for the gyroscopes and the magnetometers.

The trade-off between high sensitivity and high dynamic range affect the ability to have simultaneous combined applications when using the commercial off-the-shelf IMUs. Of course, this is less seen in combining applications with a smaller gradient of requirements. This is why combined applications of AR and posture analysis are easily found in the literature [35,89,90]. To keep using commercial IMU for the combined applications, a smart management algorithm that actively programs the dynamic range of the sensor with respect to the measured input would be needed. Such an algorithm would also be beneficial for detecting the motionless moments for SCG analysis as described before. As another workaround, Gupta et al. [36] managed to design the ACM that enjoys the benefit of high sensitivity while covering a high dynamic range. ACM was used to combine SCG with AR (Figure 2).

### 5.4. Sensor-Related Challenges

To employ IMUs in practice, choosing the right place within the chest area is the first question. This is especially more in the spotlight for the purpose of combined applications since changing the sensor place can impact the received signal. As shown in Figure 3, while for most of the applications, the sensors are perfectly distributed around the sternum, the neck is the preferred target for voice and swallow analysis. This gives rise to a research question: “where is the perfect position on the body from where swallow, voice and cardiopulmonary signals as well as the activity and posture are best mutually measured?”. A good starting point to consider may be the top of the manubrium where the bone tissue starts, as it can still transform vibrations of the voice and swallowing while not being prone to the additional degrees of freedom for making movements as the neck has.

Two other sensor-related issues are tied up with the name of the IMUs: sensor mass and sensor calibration. Few studies have investigated the effects of the sensor mass for the applications addressed in this survey. A study about seismocardiography by Yang and Tavassolian [15] proposed a simplified model for the mechanical coupling of the IMU to the chest wall. They set up an experiment consisting of an IMU wrapped in two different boxes with different dimensions and masses. They compared the data taken through accelerometers and gyroscopes from the two boxes. Their results showed that linear acceleration is less influenced by the differences of the two boxes than angular velocity; however, they could not completely explain these differences with their simplified model [15]. The mass loading effect is also to be deeper investigated in future studies to better determine its effects on the chest-worn IMUs.

**Figure 5:**
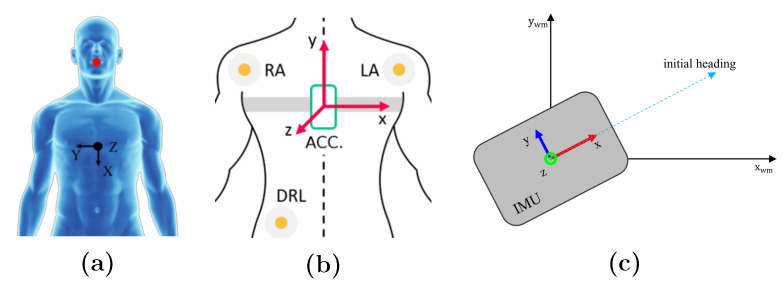
Examples of IMU coordinates alignment on body taken from the referenced studies. (**a**,**b**): IMU acceleration coordinates with respect to body axes for SCG, respectively, from [19,29]. (**c**): Calibration of the IMU pose with initial heading of the subject within the world map frame for PDR [68].

Regarding sensor calibration, it is of high importance to give special attention to perfect alignment of the sensor coordinates with the desired body axes. This is always the fundamental step of running the experiments in the referenced studies. Figure 5 shows examples of reporting sensor alignment from the referenced studies.

### 5.5. User Friendliness

While users mostly prefer the wrist site for their wearable sensors, positions with less mobility have shown to be more promising for certain applications. Zhang et al. [34] compare wrist, waist and chest for physical activity measurement and report that the participants found the chest site more acceptable than the waist site.

Therefore, it is important to keep on trying to find fashionable, user-friendly and convenient ways of producing chest-worn wearables that still provide acceptable contact for conducting the measurements. Using adhesives does not seem to be applicable for a recurring usage, and having a loosely worn strap does not meet the requirement of a sturdy contact for an artifact-free SCG. Thinking of more innovative ways such as screen printing of the PCB on the garments, adding the ability to have the sensor pierced on the skin, having the hardware as small and low-power as possible and making use of energy-harvesting techniques seems to be necessary for the future of the chest-worn inertial sensors.

## 6. Conclusions

Wearing IMUs on the chest offers a few advantages over other body positions: being in close contact with the heart and the lungs, being close to the body’s center of mass and facing more general rather than detailed movements of the body. The applications that can be taken advantage of using the chest-worn IMUs are extended thanks to the advances in signal processing and machine learning methods. In this survey, a total number of 57 studies that benefit from the chest-worn inertial sensors were screened and categorized into seven application domains, namely: *Seismocardiography*, *Activity Analysis*, *Posture Analysis*, *Localization*, *Voice Analysis*, *Swallow Analysis* and *Context Retrieval*.

The referenced studies were investigated to extract the following information out of them: the sensors used, their placement details, the validation methods and the hardware details of their sensing-processing architecture. The investigations show meaningful correlations within individual application domains; however, diversity of the requirements among the applications is a challenge in the way of benefiting from multiple applications simultaneously. Moreover, noise and artifact removal is still a significant issue to address, especially when it comes to combining the applications or maintaining the user-friendliness of the worn hardware.

## Figures and Tables

**Table 1:** List of the recent relevant work together with their scopes.

Reference	Year	Target Wearable	Scope
Cosoli et al. [18]	2020	Wrist- and chest-worn devices	Analysis of the accuracy and metrological characteristics of wearable devices for the purpose of activity monitoring.
Taebi et al. [7]	2019	Chest-worn SCG sensors	Advances in measurement and signal processing methods for the purpose of Seismocardiography.
Kröger et al. [4]	2019	Accelerometers carried out	Possible applications of the acceleration data and the privacy concerns associated with them.
Current survey	2021	Chest-worn inertial sensors	Existing applications of inertial sensors worn on the chest and their associated methods.

**Table 3:** Inertial sensors and their sensitivity versus their specific applications in the referenced studies. Preceding numbers in “Type” column reveal degree of freedom.

	Sensor	Manufacturer	Type	Sensitivity	Use Case
IMU	ICM-20602	TDK-InvenSense	6-MEMS-IMU	131 LSB/(dps) 16,384 LSB/g	SCG [54]
	MPU-6050	TDK-InvenSense	6-MEMS-IMU	131 LSB/(dps) 16,384 LSB/g	Swallow detection [47]
	MPU-9250	TDK-InvenSense	9-MEMS-IMU	131 LSB/(dps) 16,384 LSB/g 0.6 μT/LSB	SCG [20,29,30] PDR [69]
	LSM9DS0	STMicroelectronics	9-MEMS-IMU	8.75 mdps/LSB 0.061 mg/LSB 0.08 mgauss/LSB	SCG [80]
	LSM6DS3	STMicroelectronics	6-MEMS-IMU	4.375 mdps/LSB 0.061 mg/LSB	SCG [24]
Accelerometer	ADXL327	Analog Devices	3-MEMS-xl	420 mV/g	Swallow detection [48,71,72,73,75]
	ADXL345	Analog Devices	3-MEMS-xl	256 LSB/g	PDR [70]
	ADXL354	Analog Devices	3-MEMS-xl	400 mV/g	SCG [23,59]
	ADXL355	Analog Devices	3-MEMS-xl	256,000 LSB/g	SCG [19]
	MMA8451Q	NXP Semiconductors	3-MEMS-xl	4096 counts/g	SCG [55,57]
	LIS344ALH	STMicroelectronics	3-MEMS-xl	Vdd/5 V/g	SCG [58]
	1521	Silicon Designs	1-MEMS-xl	2000 mV/g	SCG [60]
	BMA280	Bosch Sensortec	3-MEMS-xl	4096 LSB/g	SCG [26]
	BU-27135-000	Knowles Electronics	1-xl	−45.0 dB re 1V/g	Voice analysis [41,45,46]
	ADXL330	Analog Devices	3-MEMS-xl	300 mV/g	Voice analysis [42] EE estimation [11] AR [64]
	ADXL210	Analog Devices	2-MEMS-xl	100 mV/g	AR [63]
Gyro.	ITG-3200	TDK-InvenSense	3-MEMS-gyr	14.375 LSB/(dps)	PDR [70]
	MAX21000	Maxim Integrated	3-MEMS-gyr	960 digit/(dps)	SCG [55]
	ADXRS300	Analog Devices	1-MEMS-gyr	1 (dps)/V	AR [63]
Mg.	HMC5883L	Honeywell	3-MEMS-mg		PDR [70]
				**Use Case**	
Platform	Smartphone		9-IMU	Postural control [39] ER from gait analysis [51] Age estimation from gait analysis [53]	
	Opal	APDM	9-IMU	Postural control in MS patients [39] Age [53], gender and height estimation [76]	
	BioPatch	ZephyrLife	3-xl	Posture detection for sleep analysis [37]	
	Physilog system	GaitUp	6-IMU	Postural control [38] AR [66]	
	GT3X+	Actigraph	3-xl	Physical activity measurement [34]	
	NGIMU	x-io Technologies	9-IMU	PDR and indoor positioning [68]	
	Shimmer	Shimmer	9-IMU	VAD [43,44] Detection of mood changes from VAD [44] AR [32,33,61,62,65] AR and fall detection [35]	

Note: xl: Accelerometer; gyr: Gyroscope; mg: Magnetometer.

**Table 4:** Inertial sensors and validation methods used in the referenced studies versus their application.

		Seismocardiography	Activity Analysis	Posture Analysis	Localization	Voice Analysis	Swallow Analysis	Context Retrieval
	Total references screened	20	13	4	3	5	7	6
Inertial Sensor	Accelerometer							
	uni-axial	25%				60%		
	bi-axial						14.3%	
	tri-axial	70%	100%	100%	100%	40%	85.7%	100%
	Gyroscope							
	uni-axial	10%						
	tri-axial	20%	23.1%		100%			50%
	Magnetometer							
	tri-axial	5%			33.3%			
Validation Method	Electrocardiography (ECG)	80%						
	Impedance Cardiogram (ICG)	5%						
	Sphygmomanometry	5%						
	Spirometry	5%						
	Blood Pressure Cuff	5%						
	Optoelectronic Plethysmography	5%						
	Respiration Belt	5%						
	Electronic Stethoscope	5%						
	Motion Capture System		7.7%					
	Indirect Calorimetry		7.7%					
	Multiple IMUs		7.7%	25%				
	Polysomnography			50%				
	Microphone					40%		
	Glottal airflow					20%		
	Video Recordings					20%		
	Videofluoroscopy						85.7%	
	Emotion Elicitation							33.3%
	Self-reported questionnaires							50%
	Observer Assessment		76.9%	25%	100%	20%	14.3%	16.7%

**Table 5:** Processing units used for different stages in the referenced studies. ✪ Shows that the *on-body* hardware is responsible for the stage, ❈ indicates that the stage is handled by a *middleware* and ❢ shows that an off-body processing *station* handles the stage.

	Description	Sensing	Acquisition	Transmission	Storage	Preprocessing	Processing	Reference
S.1	Data are collected from the IMU **on-body** and transmitted to a **middleware** for preprocessing and storage. Data are then downloaded to a **station** for processing.	✪	✪	✪	❈	❈	❢	[28]
S.2	Data are collected from the IMU **on-body** and transmitted to a **middleware** for storage. Raw data are then downloaded to a **station** for processing.	✪	✪	✪	❈	❢	❢	[38]
S.3	A data acquisition **middleware** collects and stores the IMU data. Raw data are then downloaded to a **station** for processing.	✪	❈		❈	❢	❢	[45,46,63]
S.4	IMU data are collected by a data acquisition **middleware** and directly transmitted to a **station** for storage and any processing.	✪	❈	❈	❢	❢	❢	[23,36,48,58,59,60,71,72,73,75]
S.5	IMU data are collected **on-body** and directly transmitted to a **station** for storage and any processing.	✪	✪	✪	❢	❢	❢	[10,19,29,30,33,35,47,54,62,65,67,68,74]
S.6	IMU data are collected and stored **on-body**. Data are then downloaded to a **station** for any processing.	✪	✪		✪	❢	❢	[24,26,34,37,39,41,55,57,61,66]

Note: ‘S.’ stands for “Setup” | ✪: On body / ❈: Middleware / ❢: Station.

**Table 6:** On-body processor hardware used in the referenced studies along with the use case and how the unit was powered.

Model	Manufacturer	Description	Use Case	Power Source
**Off-the-shelf boards**				
Uno R3 μC board	Arduino	Based on the ATmega328P (AVR RISC 8b, 32 KB ISP Flash, 1 KB EEPROM, 2 KB SRAM)	Data acquisition (1 kHz) and storage (memory card) [41]	
Leonardo μC board	Arduino	Based on the ATmega32u4 (AVR RISC 8b, 32 KB ISP Flash, 1 KB EEPROM, 2.5 KB SRAM)	Data acquisition (250 Hz) and transmission (serial) [30]	USB
Pro-Mini μC board	Arduino	Based on ATmega168 (Flash memory: 16 KB, SRAM: 1 KB, EEPROM: 512 bytes)	Data acquisition (I2C, 40 Hz) and transmission (BLE) [28]	Battery (Li-Po)
Mega μC board	Arduino	Based on ATmega2560 (Flash memory: 256 KB, SRAM: 8 KB, EEPROM: 4 KB)	Data acquisition and transmission (wireless) [70]	Battery
Raspberry Pi Zero W	Raspberry Pi	1GHz, single-core CPU, 512 MB RAM, wireless LAN and Bluetooth connectivity	Data acquisition (550 Hz) and transmission (Wi-Fi) [29]	
FRDM-KL25Z	NXP Semiconductor	Based on MKL25Z128VLK4 (Arm Cortex-M0+, 48 MHz, 128 KB flash, 16 KB SRAM)	Data acquisition (800 Hz) and storage (memory card) [57]	
CC2650STK SimpleLink	Texas Instruments	Multi-sensor board with ARM Cortex-M3 processor	Data acquisition (250 Hz) and transmission [20]	Battery (CR2032)
**Researcher-designed hardware**				
STM32F411CEY6	STMicroelectronics	Arm Cortex-M4 32b MCU+FPU, 125 DMIPS, 512 KB Flash, 128 KB RAM	Data acquisition (SPI, 800 Hz) and transmission (serial) [54]	USB
ATMEGA1284P	Microchip	AVR RISC 8b, 128 KB ISP Flash, 4 KB EEPROM, 16 KB SRAM	Data acquisition (500 Hz) and storage (memory card) [26]	Battery
MSP430	Texas Instruments	16-bit RISC CPU, up to 512 KB flash and 64 KB RAM	Data acquisition (60 Hz) and transmission (wireless) [10]	

**Table 7:** Middleware devices used in the referenced studies to handle some part of the processing chain from on-body sensor to the processing station.

Model	Manufacturer	Application
**Data Acquisition System**		
MP150	BIOPAC	Acquisition and transmission of acceleration, ECG and BCG [59];
		Acquisition and transmission of acceleration, gyration, ECG, BCG and ICG [23]
MP36	BIOPAC	Acquisition and transmission of acceleration, respiration (thoracic piezoresistive band) and ECG [58]
IX-228/S	iWorx	Acquisition and transmission of acceleration and ECG [60]
6210 DAQ	National Instruments	Acquisition and transmission of acceleration [48,71,72,73,75] and microphone [48,71,72,75]
**Smartphone**		
Nexus S	Google/Samsung	Acquisition and storage of acceleration [45,46]
<not reported>	–	Gathering, storage and preprocessing signals from three IMUs [28]

**Table 8:** Machine learning methods used in the referenced studies versus application area. Acronyms used in this table: AdaBoost—Adaptive Boosting; ANN—Artificial Neural Network; CNN—Convolutional Neural Network; DNN—Deep Neural Network; GMM—Gaussian Mixture Model; k-NN—k-Nearest Neighbor; LDA—Linear Discriminant Analysis; ML—Machine Learning; MLP—Multilayer Perceptron; PCA—Principal Component Analysis; SVM—Support Vector Machine; VAE—Variational Autoencoder.

ML Method	Seismocardiography	Activity Analysis	Posture Analysis	Localization	Voice Analysis	Swallow Analysis	Context Retrieval
AdaBoost		[61]					
ANN, MLP		[33,62]					[53]
CNN	[21]						[77]
Decision Tree		[33,62]				[72]	
DNN						[71]	
GMM							[78]
k-NN	[26]	[32,33,61,64]			[43]		[44]
LDA, PCA	[28]					[74]	
Naïve Bayes		[33,62,67]			[43]	[72]	[44]
Regression Models	[23,25,56]	[11,67]	[37,38]	[68,69]		[72]	
Random Forest		[35,67]					[51,53,76,77]
SVM		[33,61,62]			[43]	[47,72]	[44,51,53]
U-Net	[59]						
VAE	[21]						

**Table 9:** Specifications of the datasets used in the referenced studies.

Dataset	Sensor details Type: Part# (Manufacturer)	Participant Statistics Total (M:F) Item (Unit): Range (mean ± SD)	Description	Use Case
Mechanocardiograms with ECG References [55,84]	3-xl: MMA8451Q (NXP) and 3-gyr: MAX21000 (Maxim) On sternum (upper chest); 2-lead ECG: ADS1293 (TI)	29 (29 : 0) Age: 23-41 (29 ± 5) Height(cm): 170–190 (179 ± 5) Weight(kg): 60–98 (76 ± 11) BMI(kg/m^2^): 18–30 (24 ± 3.00)	Mechanocardiogram recordings (3-axis accelerometer and 3-axis gyroscope signals) with ECG reference were collected from healthy subjects lying either in the supine position or on their left or right side. Sensors attached to the subjects’ sternum using double-sided tape.	SCG [16]
WESAD [85]	3-xl on lower chest and on wrist; ECG, EDA, EMG, respiration and temperature;	15 (12 : 3) Age: (27.5 ± 2.4)	WESAD database is a collection of motion (acceleration) and physiological signals from both chest and wrist of the participants for stress and affect detection. The three affective states of neutral, stress and amusement were elicited in the participants, and the signals were recorded accordingly.	Context Retrieval [77]
MHEALTH [65]	9-IMU: Shimmer (Shimmer) On chest left ankle, right wrist	10	Participants performed 12 daily living activities, including Walking, Sitting and relaxing, Standing still, Lying down, Climbing stairs, Running and Cycling. The dataset also includes 2-lead ECG recordings of the participants.	AR [65]
Combined measurement of ECG, Breathing and Seismocardiogram (CEBS) [58,86]	3-xl: LIS344ALH (ST) On chest; Piezoresistor: SS5LB (BIOPAC) On Thorax; 2-lead ECG	17 (11 : 6) Age: (24.7 ± 3.9) BMI(kg/m^2^): (24.7 ± 3.9)	ECG, respiration and acceleration of 17 subjects in supine position were collected. First the basal state of the subjects was recorded for 5 min. Then, the subjects listened to music for approximately 50 min. Finally, all 5 additional minutes of data were recorded from the subjects after the music ended.	SCG [21,22]
Daily Life Activities (DaLiAc) [61]	6-IMU: Shimmer (Shimmer) On chest right hip, left ankle, right wrist	23 (13 : 10) Age: (27 ± 7) BMI(kg/m^2^): (24.0 ± 3.5)	A total of 23 healthy subjects performed 13 daily life activities: Sitting, Lying, Standing, Washing dishes, Vacuuming, Sweeping, Walking outside, Ascending stairs, Descending stairs, Treadmill running (8.3 km/h), Bicycling (50 watt), Bicycling (100 watt) and Jumping rope chosen according to their MET values	AR [32]

## Abbreviations

The following abbreviations are used in this manuscript:

ACM	Accelerometer Contact Microphone
AdaBoost	Adaptive Boosting
ADC	Analog to Digital Converter
AI	Artificial Intelligence
ANN	Artificial Neural Network
AO	Aortic valve Opening
AR	Activity Recognition
ASR	Automatic Speech Recognition
BCG	Ballistocardiogram
BP	Blood Pressure
CAD	Coronary Artery Disease
CEBS	Combined measurement of ECG, Breathing and Seismocardiogram
CNN	Convolutional Neural Network
CV	Cross Validation
DaLiAc	Daily Life Activities
DAQ	Data Acquisition
DB	Database
DNN	Deep Neural Network
DoF	Degree of Freedom
ECG	Electrocardiography
EDA	Electro-dermal Activity
EE	Energy Expenditure
EMG	Elegtromyogram
ER	Emotion Recognition
GCG	Gyrocardiography
GMM	Gaussian Mixture Model
GNSS	Global Navigation Satellite System
gyr	Gyroscope
HF	Heart Failure
HR	Heart Rate
HRV	Heart Rate Variability
I2C	Inter-Integrated Circuit
IBI	Inter-beat Interval
ICG	Impedance Cardiogram
IMU	Inertial Measurement Unit
k-NN	k-Nearest Neighbor
LDA	Linear Discriminant Analysis
LVET	Left Ventricular Ejection Time
MCU	Microcontroller Unit
MEMS	Micro-electro-mechanical System
MET	Metabolic Equivalent of Task
mg	Magnetometer
ML	Machine Learning
MLP	Multilayer Perceptron
MS	Multiple Sclerosis
PCA	Principal Component Analysis
PCB	Printed Circuit Board
PCG	Phonocardiogram
PEP	Pre-Ejection Period
PDR	Pedestrian Dead Reckoning
PPG	Photoplethysmogram
RMSE	Root Mean Square Error
RSSI	Received Signal Strength Indicator
SCG	Seismocardiography
SPI	Serial Peripheral Interface
SoC	System on Chip
SVM	Support Vector Machine
TRL	Technology Readiness Level
VAD	Voice Activity Detection
VAE	Variational Autoencoder
XGBoost	Extreme Gradient Boosting
xl	Accelerometer

## Appendix A. Referenced Studies

**Table A1:** List of the referenced studies with their applications and measurement methods. Preceding numbers in “Sensor” column reveal degree of freedom.

Reference	Sensor	Worn on	Fixation	Application
**Seismocardiography**				
Gupta et al. [36]	3-ACM: Own fabrication	Midsternum	Elastic strap over skin	SCG for heart and respiration parameters and body motion
Yu and Liu [54]	3-xl: ICM-20602 (TDK-InvenSense)	Left side of the sternum and right side of the back	Strap over skin	Motion artifact removal from SCG for heartbeat detection
Hersek et al. [59]	3-xl: ADXL354 (Analog Devices) and a modified weighting scale for BCG measurement [91]	Midsternum	Kinesio tape	Mapping SCG to BCG
Sieciński et al. [16]	Used DB: Mechanocardiograms with ECG References [55,84]			HRV analysis
Mora et al. [21]	Used DB: CEBS [58,86]			SCG for heartbeat detection and IBI estimation
Choudhary et al. [22]	Used DB: CEBS [58,86]			SCG for detection of AO-peaks
Ahmaniemi et al. [24]	3-xl: LSM6DS3 (STMicroelectronics) and PCG	Heart apex	Pocket of a belt	SCG for estimation of HR, PEP and LVET
Cocconcelli et al. [19]	3-xl: ADXL355 (Analog Devices)	Midsternum		SCG for heartbeat detection
Shandhi et al. [23]	3-xl: ADXL354 (Analog Devices) and 3-gyr: QGYR330HA (Qualtre)	Midsternum		SCG for PEP estimation
Dehkordi et al. [25]	1-xl: ultra low-frequency piezoelectric crystal accelerometer (Seismed Instruments)	Xiphoid process		SCG to identify patients with CAD
Hernandez and Cretu [20]	1-gyr: MPU-9250 (TDK-InvenSense)	Xiphoid process	Elastic fabric belt	Estimation of HR during sleep
D’Mello et al. [30]	3-xl: MPU-9250 (TDK-InvenSense)	Xiphoid process	Strap	Cardio-respiratory analysis
Kaisti et al. [55]	3-xl: MMA8451Q (NXP Semiconductors); 3-gyr: MAX21000 (Maxim Integrated);	Midsternum	Double-sided tape	SCG for heartbeat detection
Sørensen et al. [60]	1-xl × 2: 1521 (Silicon Designs)	Xiphoid process and fourth intercostal space	Double adhesive tape over skin	Relating SCG to ultrasound images
Inan et al. [26]	3-xl: BMA280 (Bosch Sensortec)	Midsternum	Adhesive-backed gel electrodes	Identification of heart failure states
Selvaraj and Reddivari [56]	3-xl and ECG and PPG	Left side of the chest	Adhered over skin	BP measurement
García-González et al. [58]	3-xl: LIS344ALH (STMicroelectronics)	Chest		Heartbeat detection and RR time series analysis
Skoric et al. [29]	3-xl-gyr: MPU-9250 (TDK-InvenSense)	Xiphoid process	Double-sided tape	Respiration rate and volume
Cesareo et al. [28]	9-IMU: LSM9DS0 (STMicroelectronics) [80]	Chest (right side), abdomen and coccyx		Respiration analysis
Jafari Tadi et al. [57]	3-xl: MMA8451Q (NXP Semiconductor)	MidSternum	Elastic strap	Gating nuclear imaging based on cardio-respiratory analysis
**Activity Analaysis**				
Barbareschi et al. [67]	3-xl	Chest (manubrium)	Double-sided tape	Evaluating transfer skills of wheelchair users
Nazarahari and Rouhani [66]	3-xl: Physilog system (GaitUp)	Chest (midsternum)	Medical tape	AR
Zhang et al. [34]	3-xl: GT3X+ (Actigraph)	Chest (xiphoid process), wrist and waist	A soft nylon necklace underneath clothes	Physical activity measurement
Awais et al. [32]	Used DB: DaLiAc dataset [61]			AR with 13 classes
Altini et al. [11]	3-xl: ADXL330 (Analog Devices)	Chest, Thigh, Ankle, Wrist and Waist	Elastic strap	EE estimation
Banos et al. [65]	3-xl: Shimmer	Chest, Ankle and Wrist	Elastic strap	AR with 12 classes
Gao et al. [33]	3-xl: Shimmer	Chest (midsternum), under-arm, waist and thigh	Fitted into a garment worn over other clothes	AR with 5 classes
Gjoreski et al. [35]	3-xl: Shimmer	Chest (xiphoid process) and thigh	Elastic Velcro straps	AR with 6 classes and fall detection
Leutheuser et al. [61]	3-xl-gyr: Shimmer	Chest (midsternum), wrist, hip and ankle	Embedded in special clothes	AR with 13 classes
Cleland et al. [62]	3-xl: Shimmer	Chest (xiphoid process), wrist, lower back, hip, thigh and foot	Elastic strap and holster over clothes	AR with 7 classes
Godfrey et al. [63]	3-xl: ADXL210 (Analog Devices); 3-gyr: ADXRS300 (Analog Devices)	Midsternum	Strap over clothes	AR with 8 classes
Atallah et al. [64]	3-xl: ADXL330 (Analog Devices)	Chest (midsternum), ear, arm, wrist, waist, knee and ankle	Strap over clothes	AR with 5 classes
**Posture Analysis**				
Hsieh and Sosnoff [39]	3-xl: Smartphone	Midsternum	Held along the sternum with hand	Postural control in MS patients
Reynard et al. [38]	3-xl: Physilog system (GaitUp)	Midsternum	Belt over clothes	Medical approach (postural control)
Razjouyan et al. [37]	3-xl: BioPatch ZephyrLife	Midsternum	Adhesive patch over skin	Posture detection for sleep analysis
Nam et al. [10]	3-xl	Xiphoid process	Belt over clothes	Posture detection for sleep analysis
**Localization**				
Lu et al. [68]	3-xl-gyr and barometer: NGIMU (x-io Technologies)	Xiphoid process	Stretching strap over clothes	Indoor positioning (PDR)
Tateno et al. [69]	3-xl-gyr: MPU-9250 (TDK-InvenSense) and RSSI	Xiphoid process	Strap over clothes	Indoor positioning (PDR)
Hu et al. [70]	3-xl: ADXL345 (Analog Devices); 3-gyr: ITG-3200 (TDK-InvenSense); 3-mg: HMC5883L(Honeywell)	Chest	Velcro belt over clothes	Indoor positioning (PDR)
**Voice Analysis**				
Dubey et al. [41]	1-xl: BU-27135-000 (Knowles Electronics)	Neck	Double sided tape and Blenderm tape over skin	VAD (medical approach)
Mehta et al. [45]	1-xl: BU-27135-000 (Knowles Electronics)	Neck	Hypoallergenic double-sided tape over skin	Measurement of vocal functions (medical approach)
Mehta et al. [46]	1-xl: BU-27135-000 (Knowles Electronics)	Neck	Hypoallergenic double-sided tape over skin	Measurement of vocal functions (medical approach)
Vitikainen et al. [42]	3-xl: ADXL330 (Analog Devices)	Neck	Adhesive tape over skin	Voice onset detection (medical approach)
Matic et al. [43]	3-xl: Shimmer	Midsternum	Elastic strap over skin	VAD
**Swallow Analysis**				
Khalifa et al. [71]	3-xl: ADXL327 (Analog Devices) contact microphone	Anterior neck overlying the cricoid cartilage		Swallow detection in patients
Donohue et al. [72]	3-xl: ADXL327 (Analog Devices) contact microphone	Anterior neck at the level of the cricoid cartilage	Adhesive tape	Swallow comparing between healthy people and Neurodegenerative patients
Donohue et al. [73]	3-xl: ADXL327 (Analog Devices)	Anterior neck	Adhesive tape	Investigating swallowing vibrations
Steele et al. [74]	2-xl	Anterior neck, below the palpable lower border of the thyroid cartilage	Single-use, disposable fixation unit	Swallow analysis for dysphagia detection
He et al. [75]	3-xl: ADXL327 (Analog Devices) and contact microphone	Anterior neck over the palpable arch of the cricoid cartilage	Double-sided tape	Investigating swallowing vibrations
Li et al. [47]	3-xl: MPU-6050 (TDK-InvenSense) [92] and PPG	Throat (cricoid cartilage)	Medical adhesive tape over skin	Swallow detection
Zahnd et al. [48]	3-xl: ADXL327 (Analog Devices)	Throat (cricoid cartilage)	Adhesive tape over skin	Investigating swallowing vibrations
**Context Retrieval**				
Hashmi et al. [51]	3-xl-gyr: Smartphone	Midsternum	Elastic strap over clothes	ER from gait analysis with 6 classes
Riaz et al. [53]	3-xl-gyr: Smartphone and Opal (APDM)	Midsternum	Elastic strap over clothes	Age estimation from gait analysis
Uddin and Canavan [77]	Used DB: WESAD [85]			Stress and Meditation Detection
Riaz et al. [76]	3-xl-gyr: Opal (APDM)	Chest (xiphoid process), wrist, ankle and lower back	Elastic strap over clothes	Estimation of age, gender and height from gait analysis
Matic et al. [44]	3-xl: Shimmer	Midsternum	Elastic strap over skin	Correlation of VAD and mood changes
Vural et al. [78]	3-xl	Midsternum	Strap over clothes	Biometric verification
